# Successful expanded clinic network collaboration and patient tracing for retention in HIV care

**DOI:** 10.1186/s12981-022-00476-x

**Published:** 2022-12-05

**Authors:** Shivani Bhatt, Mellissa Bryant, Helen Lau, Ban-Kiem Tee, Beng Eu, Jessica O’Bryan, Ian Woolley, Jeni Mitchell, Alan Street, Sheranne Dobinson, Nicholas Medland, Judy Lamb, Andrew Mahony, Adrian Tramontana, Lyn-Li Lim, Amanda Wade, Christine Roder, William Mitchell, Christopher Sherman, Fran Bramwell, Craig Aboltins, Siaw Hui Wong, Maxine Giourouki, Jennifer F Hoy, James H McMahon

**Affiliations:** 1 The Alfred Hospital, Melbourne, Australia; 2 Prahran Market Clinic, Melbourne, Australia; 3 Centre Clinic, Melbourne, Australia; 4 Access Health , Melbourne, Australia; 5 Monash Health, Melbourne, Australia; 6 Melbourne Health, Melbourne, Australia; 7 Melbourne Sexual Health Centre, Melbourne, Australia; 8 Bendigo Health, Bendigo, Australia; 9 Austin Health, Melbourne, Australia; 10 Western Health , Melbourne, Australia; 11 Eastern Health , Melbourne, Australia; 12 Barwon Health, Geelong, Australia; 13 Northside Clinic , Melbourne, Australia; 14 Armadale Family Practice , Melbourne, Australia; 15 Health Works Co Health , Melbourne, Australia; 16 Northern Health , Melbourne, Australia; 17 St Vincent’s Hospital,, Melbourne, Australia; 18 Monash University, Melbourne, Australia

**Keywords:** HIV, Retention in care, Lost to follow-up, Cascade of care, Intervention study, Clinical outcomes

## Abstract

**Background:**

There are more than 7,800 people living with human immunodeficiency virus (HIV) in Victoria, Australia. Crucial in maximising the individual and population level benefits from antiretroviral therapy (ART) is understanding how to achieve patient retention in care and the factors that drive it. This study was an expansion of a 2015 assessment of HIV-care retention in Victoria, which sought out to determine whether the inclusion of a broader range of HIV-healthcare sites would yield more accurate estimates of retention in HIV-care. We aimed to improve our understanding of HIV-care retention in Victoria, Australia, identify people living with HIV (PLHIV) with unknown outcomes, and attempt to re-engage PLHIV in care.

**Methods:**

A network of 15 HIV-care sites was established in Victoria, Australia across diverse care settings which ranged from low-caseload rural sites to high-caseload metropolitan GP clinics and hospitals. Individuals who had an HIV viral load (VL) performed in both calendar years of 2016 and 2017 were classified as retained in care. Individuals with a VL test in 2016 but not in 2017 were considered to potentially have unknown outcomes as they may have been receiving care elsewhere, have disengaged from care or died. For this group, an intervention of cross-referencing partially de-identified data between healthcare sites, and contact tracing individuals who still had unknown outcomes was performed.

**Results:**

For 5223 individuals considered to be retained in care across 15 healthcare sites in the study period, 49 had unconfirmed transfers of care to an alternative provider and 79 had unknown outcomes. After the intervention, the number of unconfirmed care transfers was reduced to 17 and unknown outcomes reduced to 51. These changes were largely attributed to people being reclassified as confirmed transfers of care. Retention in care estimates that did not include the patient outcome of confirmed transfer of care ranged from 76.2 to 95.8% and did not alter with the intervention. However, retention in care estimates which considered confirmed transfers and those that re-entered care at a new site as retained in care significantly increased across five of the sites with estimates ranging from 80.9 to 98.3% pre-intervention to 83.3–100% post-intervention. Individuals whose outcomes remained unknown post-intervention were more often men who have sex with men (MSM) when compared to other categories (person who injects drugs (PWID), combined PWID/MSM, men who identify as heterosexual or unknown) (74.5% vs. 53.5%, [p = 0.06]) and receiving ART at their last HIV-care visit (84.3% vs. 67.8% [p = 0.09]).

**Conclusion:**

This study confirmed high retention in HIV-care and low numbers of people disengaged from HIV-care in Victoria. This was demonstrated across a larger number of sites with varying models of care than a prior assessment in 2015. These data align with national and state targets aiming for 95% of PLHIV retained in HIV-care.

## Introduction

Antiretroviral therapy (ART) has markedly improved the outcomes for people living with HIV (PLHIV), both at an individual level through reducing morbidity and mortality [1], as well as at a population level by preventing onwards transmission, in the model of treatment as prevention [2, 3]. The current Australian HIV Strategy proposes targets of 95% of PLHIV being diagnosed, 95% of people diagnosed with HIV on treatment and 95% of people on treatment having an undetectable HIV viral load (VL) by the end of 2022 [4], These targets are also repeated in the current HIV Strategy in the state of Victoria, Australia [5]. Understanding how many PLHIV who remain engaged in care and on treatment is critical to understanding if the greater population is experiencing the full benefits of high treatment coverage. In addition, identifying factors that predict disengagement from care is important to enable implementation of models of HIV care to prevent it.

Previously in Australia, individual-level data on these outcomes was lacking. In 2015 the ‘Victorian Initiative for Patient Engagement and Retention’ collaboration was established across high-caseload HIV care providers in Victoria, Australia. This collaboration sought to estimate levels of retention in care, rates of transfers of care between sites, and establish the number of people with unknown outcomes, defined as those who may have been receiving care elsewhere, have disengaged from care or died. We attempted to re-invite those people who had disengaged back into care across these six sites [6]. This report captured over 75% of PLHIV in Victoria and established methods to accurately account for people transferring care across different sites. It demonstrated high levels of retention in care and low numbers of people with unknown outcomes after an intervention to cross-reference partially de-identified data between sites and contact tracing of people with unknown outcomes. This initial study also noted that study sites were the largest HIV-care sites in the state, so there was the potential that other care sites not included in the study that had lower caseloads and fewer resources may have worse retention-in-care outcomes.

In this study we sought to expand on the previous study to better understand retention in HIV-care rates across Victoria, Australia by including a more comprehensive set of HIV-providers, including a wider geographical spread, those with lower case-burdens and more diverse models of care. This study sought to determine whether an intervention of cross-referencing patient data across a broader range of HIV-healthcare sites and contact tracing of patients with unknown outcomes would yield more accurate estimates of retention in HIV-care, and to compare these findings to those of the 2015 assessment. Another key purpose of the study is to ultimately maximise the individual and public health benefits of ART, by highlighting the importance of identifying those PLHIV with unknown outcomes, and for efforts to actively re-engage them in HIV-care.

## Methods

The clinical network of HIV providers established for the initial study in 2015 was expanded to 15 HIV-care sites across Victoria: including 6 primary care clinics with specialist general practitioners (GP) with differing caseloads of PLHIV (Prahran Market Clinic, Centre Clinic, Access Health and Community, Northside Clinic, Armadale Family Practice and Health Works Co Health), one specialist sexual health centre (Melbourne Sexual Health Centre) and eight hospitals, two of which were in regional Victoria (Alfred Health, Monash Health, Melbourne Health, Western Health, Eastern Health and Northern Health in Melbourne, Barwon Health in Geelong and Bendigo Health in Bendigo). Each site had a secure, electronic data system to identify people with a diagnosis of HIV and their history of HIV VL testing. These were via dedicated databases at the hospitals and the sexual health centre, and using the practice management software systems of the primary care sites.

The following steps were taken to identify PLHIV and classify their HIV-care status. PLHIV were identified at each of the 15 study sites by reviewing if one or more VL had been performed in the period from 1 to 2016 to 1 January 2017. Patients were classified as retained in care if they had a HIV VL performed in both 2016 and 2017 calendar years. Patients were considered to have potentially unknown outcomes if there was a VL test in 2016 but not in 2017, and then were classified using the same definitions as in the prior study [6], (Table 1).

**Table 1:** Care categories and definitions

Classification	Definition
Retained in care	Evidence of a viral load performed in both 2016 and 2017
Retained at an external site	Despite HIV viral load testing at the site, did not attend the site for HIV care plus evidence of receiving HIV-care at another site.
Shared care	Evidence that the person attends more than one site regularly for HIV-care. (e.g. Primary healthcare provider and hospital)
Died	Evidence that the person has died
Confirmed transfer	Evidence of receiving HIV-care from another HIV service provider (e.g. transfer of medical records request, medical correspondence, investigation results)
Unconfirmed transfer	Evidence of planned transfer or to continue HIV-care elsewhere, but no formal documentation to confirm that this has occurred
Unknown	No information of whether HIV-care is occurring or whether the person was alive

For individuals classified as ‘Unknown’ or ‘Unconfirmed transfer’ partially de-identified patient data was shared between participating HIV-care sites to try and confirm their outcome. Subsequently, for those who were still classified in either of these categories, staff from their HIV-care site attempted contact via telephone or email from their last known HIV care-site. If they were disengaged from care, they were invited to re-engage with HIV-care and re-classified as ‘Returned to care’ once there was evidence that the patient had attended for HIV-care. Therefore, the study intervention was: (i) cross-referencing partially de-identified data between sites followed by (ii) contact tracing of those individuals who remained after cross-referencing between sites.

Estimates of ‘Retention in care’, ‘Transfer of care (confirmed or unconfirmed)’ and ‘Unknown’ outcomes per site are expressed as proportions of the total number of people who accessed HIV-care at the site in the 2016 calendar year. Individuals considered not to have entered HIV-care (i.e. classified as ‘Retained in care at another site’) are excluded from the denominator for these estimates. McNemar’s test was used to compare categorical outcomes related to status of engagement in HIV-care (Table 2). Baseline characteristics were compared for two groups (Table 3); those who were initially considered to have ‘Unknown’ outcomes and remained unknown, who had died or had declined care were compared to the group with treatment outcomes that became known (i.e. ‘Retained in care’, ‘Confirmed transfer of care’ and ‘Return to care’).These characteristics included: age, gender, HIV acquisition risk group, patients from non-English speaking backgrounds (NESB), receiving ART at last visit, active psychiatric condition (documented or on relevant medications), VL at last visit and those enrolled in Medicare (a publicly funded healthcare scheme in Australia affording a range of health services at low or no cost). Characteristics were compared by Chi-square or Fisher’s exact test for categorical variables. For continuous variables of age and VL, the student’s T-test and Wilcoxon rank sum test were used, respectively. Statistical tests were conducted using Stata software (Version 12, College Station, TX, U.S.A.). The ethical review boards at the Alfred Hospital and Monash Medical Centre approved the study for all sites (Alfred Health HREC number 46.14, Monash Health HREC number 14142X).

## Results

We included 5223 patients in care at the 15 care sites who had VL performed in 2016 after excluding 207 who were not considered to have entered care at the original site. Of these 5223 individuals, 4735 were classified as retained in care pre-intervention. 38 had died, 84 had evidence of retention-in-care but without VL testing (e.g. evidence of prescribing or dispensing ART) but no subsequent VL testing was performed in 2017 so there was evidence of retention without VL testing, 238 were considered confirmed transfers, 49 unconfirmed transfers and 79 had unknown outcomes (Table 2). After the intervention (cross-referencing data and contact tracing) unknown outcomes reduced to 51 and unconfirmed transfers to 17 individuals. This reduction after the intervention was reflected in the increase in people who were then classified as confirmed transfers, from 238 to 271 individuals, combined with one additional death, 25 people who returned or re-engaged with care and two people who declined further care despite being contacted as part of the intervention. Changes in proportions of the unknown, confirmed and unconfirmed transfer outcomes pre and post the intervention reached statistical significance for some sites and are detailed in Table 2. Sites with statistically significant differences in estimates pre- and post-intervention largely reflected the sites caring for larger numbers of people (TMC1, SPC2, SHC1).

Retention in care estimates that did not include the patient outcome of confirmed transfer of care had the same pre- and post-intervention range of 76.2–95.8%, which did not achieve statistical significance at any site. However, retention in care estimates which considered confirmed transfers.

and those that re-engaged with HIV-care at a new site as retained in care demonstrated increased retention across five sites; three hospitals (TMC1, TMC5 and TMC7, p < 0.01), one primary healthcare site (SPC2, p < 0.01) and the sexual health centre (SHC1, p < 0.01) with overall pre- and post-intervention percentage ranges of 80.9–98.3% and 83.3–100%.

**Table 2:** Key outcomes by site (n = 5223)

Outcome											
	**Individuals in care** ^**a**^	**Unknown** ^**b**^ **n** **(%)**		**Unconfirmed transfer** ^**c**^ **n (%)**		**Confirmed transfer** ^**d**^ **n (%)**		**Retention** ^**e**^ **n (%)**		**Retention inc.** **transfer and entry to new sites** ^**f**^ **n (%)**	
**Pre or post intervention**		**Pre**	**Post**	**Pre**	**Post**	**Pre**	**Post**	**Pre**	**Post**	**Pre**	**Post**
TMC1	910	14 (1.5)	9 (0.9)#	17 (1.8)	7 (0.7)*	16 (1.7)	26 (2.8)#	90.5	90.8	92.3	93.6#
TMC2	19	1 (5.2)	0	1 (5.2)	0	2 (10.5)	3 (15.7)	78.9	78.9	89.5	100
TMC3	85	1 (1.2)	1 (1.2)	0	0	3 (3.5)	3 (3.5)	90.6	90.6	94.1	94.1
TMC4	21	0	0	1 (4.0)	0	1 (4.0)	2 (9.5)	76.2	76.2	80.9	85.7
TMC5	282	3 (1.1)	1 (0.3)	11 (3.9)	2 (0.7)	8 (2.8)	17 (6.0)#	88.7	89.4	91.5	95.4#
TMC6	28	1 (3.6)	1 (3.6)	0	0	0	0	92.9	92.9	92.9	92.9
TMC7	249	5 (2.0)	2 (1.2)	2 (0.8)	0	10 (4.0)	11 (4.4)	87.1	87.1	91.2	92.8#
TMC9	92	3 (3.3)	3 (3.3)	3 (3.3)	1 (1.1)	5 (5.4)	7 (7.6)	87.0	87.0	92.4	94.6
SPC1	490	10 (2.0)	7 (1.4)*	0	0	35 (7.1)	35 (7.1)	89.8	89.8	96.9	97.6
SPC2	1032	16 (1.5)	9 (0.8)#	0	0	38 (3.7)	39 (3.8)	94.1	94.2	97.8	98.5#
SPC3	48	0	0	0	0	1 (2.1)	1 (2.1)	95.8	95.8	97.9	97.9
SPC4	12	1 (8.3)	1 (8.3)	0	0	2 (1.7)	2 (1.7)	66.7	66.7	83.3	83.3
SPC5	59	0	0	0	0	2 (3.4)	2 (3.4)	94.9	94.9	98.3	98.3
SPC6	702	5 (0.7)	2 (0.3)	4 (0.6)	3 (0.4)	20 (2.8)	21 (2.9)	92.5	92.5	95.3	95.7
SHC1	1194	19 (1.6)	15 (1.3)#	9 (0.7)	4 (0.3)*	95 (7.9)	100 (8.4)*	88.7	89.0	96.7	97.4#
Total	5223	79	51	49	17	238	269				

**NOTES**: SPC, specialist primary care: TMC, tertiary medical centre; SHC, sexual health centre, * p < 0.05 for comparison to pre-intervention figure (McNemar’s test); #p < 0.01 for comparison to pre-intervention figure (McNemar’s test). The retention in care comparison is between the ‘Pre-Retention’ and ‘Post-retention inc. transfer and entry to new sites’

**‘Pre’** definition is based on viral load data and medical record review at the site

**‘Post’** definition is based on cross referencing data between sites and tracing people post cross referencing

^a^ individuals with at least one HIV viral load test from 1/1/2016 to 31/12/17 at the site excluding individuals who had not received HIV care at the site and were known to be in HIV care at an external site

^b^ Individuals with unknown outcomes

^c^ Individuals thought to have transferred care but no evidence in medical records to confirm that transfer occurred

^d^ Evidence in medical records that care was transferred

^e^ Individuals in care at the site or sharing with another sites as a proportion of all individuals in care

^f^ Defined as for retention but considers confirmed transfer and those that have re-entered care at a new site also retained in care

When comparing the baseline characteristics of the individuals in the two post-intervention groups, they were found to be similar in age (p = 0.13) (Table 3). Individuals whose outcomes remained unknown, were more likely to be of the male gender and to be from NESB than those who had re-entered care, 94.1% versus 89.2% (p = 0.07) and 13.7% versus 10.7% (p = 1), respectively, but results did not achieve statistical significance. Individuals whose outcomes remained unknown were more likely to be men who have sex with men (MSM) when compared to other categories (person who injects drugs (PWID), combined PWID/MSM, men who identify as heterosexual or unknown) (74.5% versus 53.5%, (p = 0.06)) and more likely to be receiving ART at last HIV-care visit with a trend towards statistical significance, 84.3% versus 67.8% (p = 0.09). People with access to Medicare had an increased proportion that returned to care, 96.4% versus 82.3% (p = 0.09), with a trend towards statistical significance.

**Table 3:** Characteristics of individuals with unknown outcomes compared for those whose outcomes remain unknown, who had died or had declined care post-intervention to those who re-entered or transferred care (n = 79)

Characteristic	People with unknown outcomes pre-intervention^a^ (n = 79)	Re-entered or Transferred care (n = 28)	Remained unknown, died or declined (n = 51)	P value
Age (± SD)	41.0 ± (12.7)	43.9 ± (12.6)	39.4 ± (12.6)	0.13
Gender (% male)	92.4%	89.2%	94.1%	0.7
Transmission risk category (% MSM)^b^	67.1%	53.5%	74.5%	0.06
Non-English speaking background^c^	12.7%	10.7%	13.7%	1
Medicare card holders^d^	87.3%	96.4%	82.3%	0.09
Receiving ART at last visit	78.5%	67.8%	84.3%	0.09
Active psychiatric condition^e^	33.8%	33.3%	34.0%	1
Viral load copies/mL (Median, IQR)	< 20 (< 20–1723)	< 20 (< 20–2295)	< 20 (< 20–1723)	1.0

**NOTES**: MSM, men who have sex with men; ART, antiretroviral therapy; UD, undetectable

Characteristics compared by χ2 test or Fisher’s exact test if cell frequencies ≤ 5 apart from age (students’ t-test) and viral load (Wilcoxon rank sum test)

^a^ Intervention of cross referencing data between sites and contact tracing for those still with unknown outcomes

^b^ MSM as compared to other categories (PWID, combined PWID/MSM, heterosexual, unknown)

^c^ English not nominated as first language or born outside of Australia and first language is not English

^d^ Holder of Medicare card that allows access to publicly funded healthcare

^e^ Receiving medication for a psychiatric condition (e.g. depression, anxiety, schizophrenia, bipolar affective disorder) or documented symptomatic psychiatric condition at last visit

## Discussion

This study which expanded on our previous study in 2015 to broaden the range of HIV-care providers to include regional and low-caseload sites, has again identified very high levels of retention in care PLHIV in Victoria, Australia. Some estimates of retention were in fact marginally higher than the estimates in 2015 [6]. We postulated that smaller and more remote sites may have lower levels of retention in HIV-care, but this was not the case. In addition, some measures of retention were in fact marginally higher than in 2015 [6]. It is also notable that the number of individuals that were considered to have disengaged from care (with unknown outcomes or had unconfirmed transfers) were consistently lower in this analysis. For example, the larger care sites in this analysis had a proportion of individuals with unknown outcomes before the intervention ranging from 0.7 to 2% as compared to 1.1–4.7% of those with unknown outcomes in 2015. The increased proportion with unknown outcomes seen in 2015 demonstrates that the intervention to cross-reference data between sites and contact trace individuals with unknown outcomes is more likely to lead to more accurate estimates of retention in HIV-care rates. In this study estimates of retention in HIV-care including confirmed transfers of care ranged from 80.9 − 98.3% which increased to 83.3- 100% following the cross-referencing data between healthcare sites intervention and contact tracing individuals with unknown outcomes. This was a numerical improvement when compared with estimates of retention in the original study, which were 85.9 − 95.8% pre-intervention and 91.4 − 98.8% post-intervention [6]. Our results align with those of high-income countries with similar quality-of-life indices and publicly-funded healthcare systems to Australia where HIV epidemics affect men, in particular MSM [7–9]. Interestingly, we found that study participants who identified as MSM were more likely to have outcomes that remained unknown post-intervention, when compared to other transmission risk categories. Other studies in the United States and Indonesia have found better or comparable rates of retention in care amongst MSM compared to other risk groups [10, 11], Data presented in this study suggests more detailed research beyond just a transmission risk category is important for determining what factors predict unknown treatment outcomes in PLHIV.

There are multiple possible explanations for maintaining or even improving such high levels of retention in care and reducing the number of people with unknown outcomes or unconfirmed transfers compared to the previous analysis. In the intervening period between the two studies there has been increasing importance placed on maintaining high levels of care engagement and the cascade of HIV care in Australia and globally [4, 5, 10, 11]. Our study highlights the need to identify PLHIV with unknown outcomes, and attempt to re-engage them in care to improve individual and population level outcomes for PLHIV. Our research may lead to HIV-healthcare centres initiating or expanding processes to track individuals missing scheduled appointments and perform their own contact tracing interventions to minimise the number of individuals with unknown outcomes. In addition, Australia maintains a publicly-funded healthcare model for PLHIV to access treatment, and there are mechanisms to obtain compassionate access to ART for those PLHIV who are ineligible for Medicare [12].

Our prior study classified individuals as retained in care if they underwent VL testing in a 9-month period, as it was agreed between the participating sites that 6 months was the longest accepted period between clinical review and VL monitoring for patients engaged in HIV-care [13]. Our follow-up study considered individuals to be retained in care if they had a single VL tested in each of 2016 and 2017. This methodological difference in definition between studies may contribute, at least in part, to the observed improvement in post-intervention retention rates in the current study. Notably, this change in definition of care retention could be considered a methodological advantage as it allows for monitoring over more time in the second follow-up period (i.e. 2017), thus increasing the chance of capturing VL testing data in this 12-month period compared with the prior study’s 9-month period. The main advantage of the current study however was the inclusion of more service providers, with different resources and frameworks for patient care [6]. The similar or even improved levels of retention seen in this current study is reassuring given the participation of smaller centres with diverse models of patient care. Another advantage of this study’s methodology to estimate retention is the use of individual-level data when compared to traditional measures relying on ecological data from observational cohort studies, such as national patterns of CD4 count testing in PLHIV [14, 15].

A limitation to our study is that not all HIV-care sites in Victoria were included in the study. Further, we did not investigate for a range of factors that could potentially predict disengagement from HIV-care. Although the proportion of PLHIV with potentially unknown outcomes who were later determined to be retained in care elsewhere in the network is high, the remaining PLHIV not able to be identified as retained in care could be receiving care elsewhere (interstate in Australia, or overseas). The number of such patients who are actually retained in care in this way in unknown, hence the measures of retention in care in this study might be an underestimate.

Risk factors such as psychiatric illness, NESB and ineligibility for Medicare were investigated again due to their significant findings in the prior study to predict disengagement from care [6] but these findings were not replicated. Previous studies have described that approximately 30% of PLHIV in Australia are not accessing treatment or appropriate care, which has been attributed to the complex interplay of emotional barriers such as stigma and fear, practical issues such as lack of knowledge and medical or service-specific barriers, among other reasons [16, 17]. Further studies are needed to investigate these factors to identify potential opportunities for interventions that may encourage retention or re-engagement in healthcare, or to prevent disengagement from HIV-care in the first place.

In conclusion, this study expanded on the work published in 2015 to include a greater cross-section of HIV-care providers and found similarly high retention rates and even lower numbers of people with unknown outcomes of PLHIV in Victoria compared to the first study. This data is reassuring as it demonstrates that retention in HIV-care in Victoria is high and at a level to meet the targets of the most recent Victorian HIV strategy aiming for 95% of people to be accessing treatment [5]. In addition, this work emphasizes the importance of our intervention to capture those who have transferred their care, in addition to implementing systems to trace individuals disengaged from care and attempt re-engagement in care to maximise the number of people in care with access to treatment. The intervention in our study also highlights the importance of keeping updated and accurate patient contact details to prevent loss to follow-up. Ongoing assessments of retention-in-care and determining outcomes for people disengaged from care, or that have transferred care will be critical to map a path to HIV elimination that maximises the number of people in care and able to receive effective ART for individual and public health benefits.

## Data Availability

Not applicable.
